# A population-based study of neonatal mortality and maternal care utilization in the Indian state of Bihar

**DOI:** 10.1186/1471-2393-14-357

**Published:** 2014-10-17

**Authors:** G Anil Kumar, Rakhi Dandona, Priyanka Chaman, Priyanka Singh, Lalit Dandona

**Affiliations:** Public Health Foundation of India, Plot 47, Sector 44, National Capital Region, Gurgaon, 122002 India; Institute for Health Metrics and Evaluation, University of Washington, Seattle, WA USA

**Keywords:** Bihar, India, Inequality, Maternal care, Neonatal mortality, Postnatal care

## Abstract

**Background:**

A substantial reduction in neonatal deaths is required in India to meet the Millennium Development Goal of a two-thirds reduction in child mortality by 2015. We report neonatal mortality estimates and utilisation of maternal care in the Indian state of Bihar.

**Methods:**

A representative population-based sample of 14,293 women who had a live birth in the last 12 months based on multistage sampling from all 38 districts of Bihar was selected for interview in early 2012. We estimated neonatal mortality rate and its associations using multiple logistic regression, assessed maternal care coverage and its inequality by wealth index, and retention of mothers in the health system for the full sequence of maternal care services.

**Results:**

Neonatal mortality rate for Bihar was 32.2 (95% confidence interval [CI] 27.6-36.8) per 1,000 live births. Postnatal care related variables were significantly associated with neonatal deaths – no delayed bathing of new born (odds ratio [OR] 3.45, 95% CI 2.47-4.81) and no kangaroo care immediately after birth (OR 2.20, 95% CI 1.49-3.25). History of maternal complications and delivery in a private sector health facility had nearly twice the odds of neonatal death; the latter was driven by the very high neonatal mortality associated with private facility delivery in the lower two wealth index quartiles. A pattern of mass deprivation was seen for coverage of 4 or more ANC visits, health facility delivery and postnatal care for the same woman, with only 5.2% of women receiving this overall; this coverage was low for the highest wealth index quartile as well at 12.2%. Coverage of 4 or more ANC visits was 7.4% and 27.7% in the lowest and the highest wealth quartiles, respectively. Giving birth in a health facility was reported by 49.5% of women in the lowest wealth index quartile and by 77.7% in the highest quartile. Only 21.2% women reported post-natal care within 2 weeks of delivery in the lowest wealth index quartile, and 42.2% in the highest quartile.

**Conclusions:**

Neonatal mortality continues to be relatively high in Bihar, and the utilization of maternal care very low and inequitable. Interventions need to address these deficiencies.

**Electronic supplementary material:**

The online version of this article (doi:10.1186/1471-2393-14-357) contains supplementary material, which is available to authorized users.

## Background

Globally, a remarkable reduction in child mortality before the age of 5 years has occurred in recent years [1, 2]. However, most of these reductions are a result of lives saved after the first 4 weeks of life, with little reduction in the risk of death in the neonatal period. A substantial reduction in neonatal deaths is required to meet the Millennium Development Goal (MDG) 4 of a two-thirds reduction in child mortality by 2015, thereby, making the reduction in neonatal deaths a major public health priority [3]. The Global Burden of Disease Study 2013 trends suggests that unless further progress is made to reducing neonatal mortality, the proportional contribution of neonatal deaths to the under-5 deaths would increase [2].

India accounts for approximately a quarter of all global neonatal deaths, and is currently off-track to meet the MDG 4 [2, 4–6]. It is estimated that 40% of all under-five deaths are in neonates, and nearly 40% of these neonatal deaths occur on the first day of life and nearly three-fifths during the first 3 days [7–9]. For India, with a population of about 1.3 billion, it is important to have relevant data at the sub-national levels, including improved estimates and causes of infant, neonatal and under-5 child mortality, which would help direct child survival resources appropriately, particularly in the poor and populous states of India that account for a large proportion of child deaths [7, 10, 11]. India also scores poorly on equity in child health, with a child born to poor parents several times more likely to die than a child born to rich parents [6]. Furthermore, important linkages exist between access to maternal and child health care services and neonatal mortality [8, 10, 12–15]. The MDG 5 is aimed at improving maternal health [3], for which data at the sub-national levels are also needed as the national level data often mask inequities within the country [10, 12, 13, 16].

This paper is based on data collected in the baseline household survey as part of the evaluation of the Ananya program in the Indian state of Bihar. This is a five-year program funded by the Bill and Melinda Gates Foundation with the long-term goal of reducing maternal, new born, and child mortality; fertility; and under nutrition rates in Bihar [17]. Bihar is one of the most populous and poorest states of India which is located in the north of the country. In this paper, we report neonatal mortality estimates and utilization of maternal health care as well as its inequality in Bihar, with the aim of highlighting the issues that need to be addressed to improve neonatal survival and maternal care.

## Methods

The state of Bihar had a population of 104 million in 2011, with 11% urban [18]. This baseline survey is part of the Ananya evaluation, which was approved by the institutional ethics committee of the Public Health Foundation of India and by the Health Ministry’s Screening Committee at the Indian Council of Medical Research. A multi-stage sampling design was used to obtain a representative sample of live births in the last 12 months from all 38 districts of Bihar. We estimated a total target sample requirement of 15,390 live births, assuming a 10% refusal rate in the survey. This sample would have 80% power at the 95% confidence level to detect a drop in neonatal mortality rate of 6 per 1,000 live births or more during the intervention period from the baseline estimate of 32 per 1,000 live births in 2011.

Each district in Bihar is divided into 5–27 administrative blocks. We first stratified the total 534 blocks of Bihar into two groups, those with only rural population and those with rural and urban population. We then sampled 342 blocks randomly from the two strata in proportion to the overall size of each stratum. We then sampled a total of 1,017 clusters from the sampled blocks using simple random sampling without replacement, which included 772 rural clusters and 245 urban clusters, the vast majority having 75–150 households each. The rural clusters were a village or part of a village based on Census 2001 [19], and the urban clusters were based on the National Sample Survey Organization urban sampling frame of 2007–2010.

All the households in each sampled cluster were enumerated to identify live births or still births in the last 12 months. A household was defined as people eating from the same kitchen. A detailed account of pregnancy outcomes in the last 12 months from the date of interview was assessed in each household. The preferred respondent for this information was the woman who had a pregnancy outcome in the last 12 months. In case this woman was not available, another woman of reproductive age in the household was asked for this information, and in the absence of this an older female in the household was asked. To ensure that no pregnancy outcome was missed during enumeration, we obtained data for the last 13 months from the date of enumeration but considered pregnancies with outcomes for the last 12 months for this analysis. Pregnancy outcome data were also collected for women who had died due to pregnancy-related causes. A birth was considered live if the new born had breathed or cried or moved at birth [20]. The date of birth and sex was recorded for live births.

All women who were identified in enumeration as having had a live birth in the last 12 months were considered eligible for a detailed interview. Forty interviewers with experience in conducting health surveys were trained to use a structured questionnaire for confidential interviews in the local language to document information about socio-demographic background, birth history, and utilisation of maternal and child care services for the most recent birth. The data collection by interviewers was monitored by supervisors. At least three attempts were made to reach the eligible mothers during the time period of data collection in a particular cluster which was generally three days. After the initial round of data collection in all clusters an addition attempt was made at the end of the survey to reach eligible women who could not contacted earlier. Data were collected from January to April 2012. Written informed consent was obtained for participation in the survey. Illiterate persons provided the right thumb impression in lieu of signature for consent. Data were entered directly in a computer by the interviewers, which were scrutinized to detect and correct errors. About 30% of the data were collected by the interviewers under direct supervision and an additional 5% of the interviews were checked by the supervisors by visiting the respondent again. Data were analysed using STATA 11.2 software (StataCorp, USA).

Neonatal death was defined as death occurring within the first 28 days of life [21, 22]. Neonatal mortality rate (NMR) per 1,000 live births is reported for the state, and for the north and south zones of the state. The north zone is relatively less developed which includes 21 districts that are north of the Ganges river, and the south zone has the remaining 17 districts [23]. Appropriate sample weights, based on over- or under-representation of certain population segments in the sample vis-à-vis the actual distribution, were used for estimating NMR and the 95% confidence intervals are reported.

We considered neonatal deaths for which detailed interview was available for multiple logistic regression in order to assess the associations of neonatal mortality with maternal age at birth, maternal education, household wealth index, maternal complication during pregnancy, sex of the child, four or more antenatal visits, consumed 90 or more iron folic acid (IFA) tablets during pregnancy, received two or more tetanus injections during pregnancy, place of delivery, delayed bathing of new born - (more than 2 days after birth), early breast feeding of new born (breastfeeding immediately or within 1 hour of birth), new born receiving Kangaroo care (skin to skin contact) immediately after birth, and postnatal care within 2 weeks. The household wealth index was calculated as in the National Family Health Survey [24]. On exploring the socioeconomic distribution of Bihar it appeared that grouping the wealth index score in quartiles would best capture the differences in socioeconomic groups for the variables in our analysis, therefore we used this grouping in this paper. In the logistic regression model, the effect of each category of a multi-categorical variable was assessed by keeping the first or the last category as reference, and all the variables were introduced simultaneously into the model. The odds ratios (OR) are presented with 95% confidence interval (CI).

We assessed the utilization of maternal heal care and explored the patterns of inequality in coverage of each component of maternal health care using a previously described approach [15, 25, 26]. The patterns of inequality in utilization of four or more ANC visits, institutional delivery, postnatal care within 2 weeks of delivery, and all of these combined (complete maternal care), were assessed by stratifying the mothers into wealth index quartiles based on their household wealth index score. Finally, we examined the retention of mothers in the health system across the three levels of maternal care – ANC, institutional delivery and postnatal care within two weeks – by the wealth index quartile. Retention was considered as being in the system for the three levels of maternal care, and for this assessment we considered those mothers who had utilized the health system for at least one ANC visit as ANC is the starting point for maternal care services.

The checklist for how this manuscript followed the STROBE guidelines for reporting observational research is shown in Additional file 1.

## Results

In the 110,094 households enumerated in the sampled clusters, 14,293 women reported at least one live birth in the last 12 months, with a total of 14,847 live births. During the enumeration, 409 neonatal deaths were identified with these children having died up to 27 days of birth. The estimated NMR for the state of Bihar was 32.2 per 1,000 live births (95% CI 27.6-36.8) (Table 1). The point estimate for NMR in the relatively less developed north zone (34.7 per 1,000 live births) of Bihar was 25% higher than for the south zone (27.7 per 1,000 live births), but this difference was not statistically significant (p = 0.18).

**Table 1:** **Estimated neonatal mortality rate in in the Indian state of Bihar**

	Total live births*	Neonatal deaths	Neonatal mortality rate per 1,000 live births (95% CI)
State	13,359	417	32.2 (27.6-36.8)
South zone^†^	5,739	163	27.7 (21.5-33.9)
North zone^‡^	7,620	254	34.7 (28.5-40.9)

*All live births aged 28 days to 12 months at the survey date.

^†^South zone: includes 17 districts south of Ganges river.

^‡^North zone: includes 21 districts north of Ganges river.

Of the 14,293 women identified with a live birth in the last 12 months, 13,069 (91.4%) participated in the detailed interview. Among the 417 women who had had a neonatal death, 261 (62.6%) participated in the detailed interview – 103 (24.7%) were out of station for an extended period, 37 (8.9%) were not available due to other reasons, and 16 (3.8%) refused participation. Among the 261 neonatal deaths available for detailed analysis (Table 2), 135 (51.7%) deaths occurred within 1 day of birth, 40 (15.3%) during 2–3 days, 35 (13.4%) during 4–7 days, and 51 (19.5%) during 8–27 days after birth. The mothers aged 15–19 years were significantly more likely to have a neonatal death (OR 3.36, 95% CI 1.53-7.39), followed by those aged 20–24 years. The odds of neonatal death were higher for women who did not consume 90 or more IFA tablets as compared with those who did (OR 1.61, 95% CI 1.06-2.45). The postnatal care related variables - no delayed bathing of new born (OR 3.44, 95% CI 2.46-4.80) and no kangaroo care immediately after birth (OR 2.22, 95% CI 1.51-3.28) were significantly associated with neonatal deaths. History of maternal complications including prolonged labor, excessive bleeding or convulsions had higher odds of neonatal death (OR 2.17, 95% CI 1.65-2.86), as did delivery at a private sector health facility versus elsewhere (OR 1.87, 95% CI 1.30-2.69). Deliveries in the private health facilities, public health facilities and at home accounted for 17.5%, 45.0% and 37.5% of the total, respectively. The public health facilities included large government hospitals and smaller hospitals or clinics, where respectively one-third and two-third of the public facility deliveries occurred. The large government hospitals generally have more advanced facilities for deliveries than the smaller hospitals or clinics. Private facility deliveries were higher in the two higher wealth index quartiles as compared with the two lower wealth index quartiles (25.8% of all deliveries versus 8.6%, p <0.001). Neonatal mortality was much higher among the private facility deliveries versus others for the two lower wealth index quartiles (5.2% versus 2.0%, p < 0.001), but not for the two higher wealth index quartiles (2.0% versus 1.7%, p = 0.33). Women who delivered in a private facility reported a higher proportion of prolonged labor (32.4% versus 20.4%, p < 0.001), excessive bleeding (14.9% versus 8.3%, p < 0.001) and convulsions (13.1% versus 8.9%, p < 0.001) as compared with women who delivered elsewhere. Women belonging to the two lower wealth index quartiles had a higher risk of neonatal death (OR 1.40, 95% CI 1.06-1.85).

**Table 2:** **Association of socio-demographic and maternal care variables with neonatal mortality using multiple logistic regression in the state of Bihar**

Variable	Categories	Total	Neonatal deaths	
		N = 13,069* (% of total)	Number (%)	Odds of neonatal death (95% CI)
Socio-demographic				
Maternal age at birth (years)	15-19	418 (3.2)	16 (3.83)	3.36 (1.53-7.39)
	20-24	4,999 (38.3)	120 (2.40)	2.20 (1.17-4.13)
	25-34	6,748 (51.6)	113 (1.67)	1.40 (0.75-2.63)
	35 or more	904 (6.9)	12 (1.33)	1.00
Maternal education	No schooling	7,725 (59.1)	161 (2.08)	1.15 (0.86-1.53)
	Any schooling	5,342 (40.9)	100 (1.87)	1.00
Wealth index	Quartiles 1 & 2	6,279 (48.0)	143 (2.28)	1.40 (1.06-1.85)
	Quartiles 3 & 4	6,790 (52.0)	118 (1.74)	1.00
Sex of child	Female	6,182 (47.3)	104 (1.68)	1.00
	Male	6,884 (52.7)	157 (2.28)	1.38 (1.07-1.78)
Care-related				
Four or more antenatal care visits	Yes	1,861 (14.2)	37 (1.99)	1.00
	No	11, 202 (85.8)	224 (2.00)	1.12 (0.77-1.63)
Received 2 or more tetanus injections during pregnancy	Yes	12,100 (92.6)	231 (1.91)	1.00
	No	969 (7.4)	22 (2.27)	1.06 (0.67-1.66)
Consumed 90 or more IFA tablets during pregnancy	Yes	1,856 (14.2)	27 (1.45)	1.00
	No	11,213 (85.8)	234 (2.09)	1.61 (1.06-2.45)
Place of delivery	Home	4,900 (37.5)	100 (2.04)	1.00
	Public facility	5,877 (45.0)	98 (1.67)	1.07 (0.79-1.44)
	Private facility	2,292 (17.5)	63 (2.75)	1.87 (1.30-2.69)
Maternal complication during pregnancy	No	8,273 (63.3)	136 (1.64)	1.00
	Prolonged labour, excessive bleeding and or convulsions	3,546 (27.1)	105 (2.96)	2.17 (1.65-2.86)
	Other complications	1,250 (9.6)	20 (1. 60)	1.02 (0.63-1.65)
Mother received postnatal care within 2 weeks	Yes	4,345 (33.3)	69 (1.59)	1.00
	No	8,724 (66.7)	192 (2.20)	1.42 (1.05-1.91)
Neonate received Kangaroo care (skin to skin contact)	Yes	2,315 (17.7)	33 (1. 43)	1.00
	No	10,728 (82.3)	228 (2.13)	2.22 (1.51-3.28)
Delayed bathing of neonate (>2 days)	No	8,198 (62.9)	216 (2.63)	3.44 (2.46-4.80)
	Yes	4,846 (37.1)	43 (0.89)	1.00
Early breast feeding of neonate (immediately/ within 1 hour)	No	7,675 (58.7)	172 (2.24)	1.29 (0.99-1.68)
	Yes	5,394 (41.3)	89 (1.65)	1.00

*Data missing: maternal education for 2; sex of child for 3; four or more antenatal care visits for 6; neonate received Kangaroo care (skin to skin contact) for 26; delayed bathing of neonate (>2 days) for 25.

CI denotes confidence interval.

**Figure 1:**
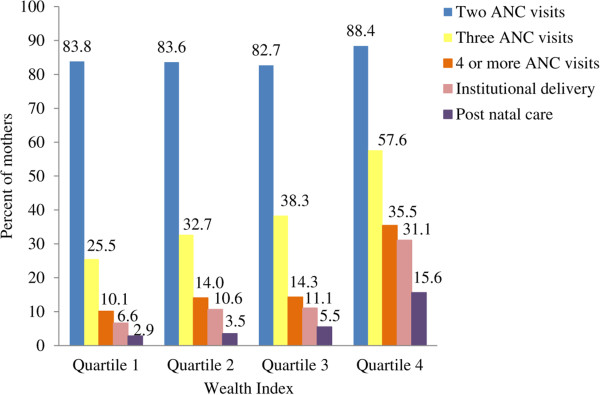
**Retention of maternal care based on the wealth index quartile for the women who had accessed the health system for at least one antenatal care visit and had a live birth in the last 12 months in the Indian state of Bihar.**

**Figure 2:**
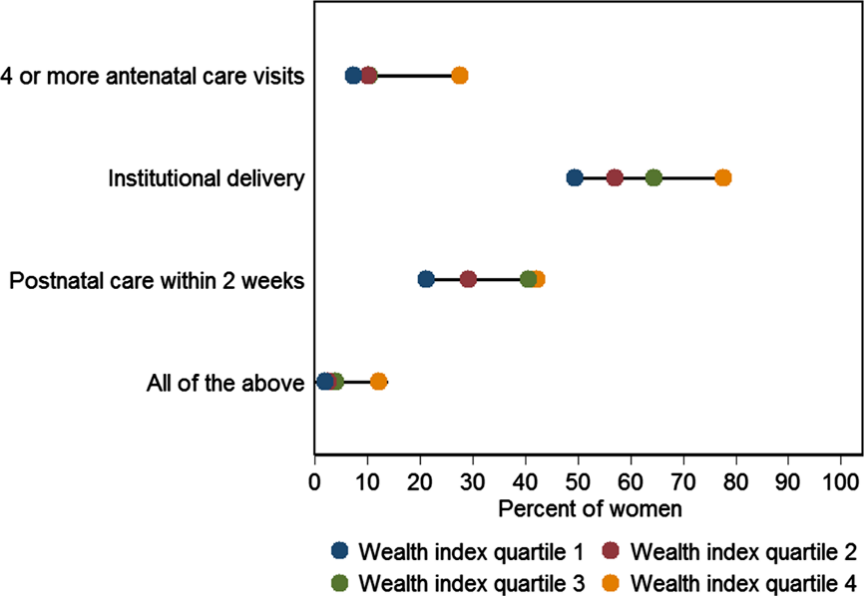
**Patterns of inequality based on wealth index quartiles for 4 or more antenatal care visits, institutional delivery, post natal care within 2 weeks of delivery, and complete maternal care for the women who had a live birth in the last 12 months in the Indian state of Bihar.**

Of the 13,069 mothers who had a live birth in the last 12 months, 11.2% did not utilized any maternal care; this group comprised of 34.5%, 30.3%, 20.4% and 14.9% women belonging to the wealth index quartiles 1, 2, 3, and 4, respectively. Of the mothers who had a live birth in the last 12 months, 14.5% utilized institutional delivery or postnatal care but did not utilize the health system for ANC. Of the total, 26.2% women did not have any ANC visit, 11.2% had only one ANC visit, 33.4% had two ANC visits, 14.9% had three ANC visits, and only 14.3% reported having completed four or more ANC visits for the birth in the last 12 months. Of the women who had any ANC visit, 59.7% had their first ANC visit within three months of pregnancy, 27.7% between 4–6 months of pregnancy, and the remaining 2.6% in the subsequent months of pregnancy. Overall, 62.2% women reported an institutional delivery and 33.3% of women reported postnatal care within two weeks of delivery and 33.8% of women received postnatal care at any time. Only 5.2% of women reported receiving all the care including four or more ANC visits, health facility delivery and postnatal care.Figure 1 shows the retention of women who had a live birth in the last 12 months for various levels of maternal services by wealth index who had accessed the health system for the first ANC visit. The proportion of women starting ANC care was not very different for the four wealth index quartiles, ranging from 72.9% to 78.3%. For all wealth index quartiles, the biggest drop in retention occurred at the ANC level between the second and third visits. Only 10.1%, 14.0% and 14.3% of all the women who had started ANC were retained for four ANC visits or more in the wealth index quartiles 1, 2, and 3, respectively and 35.5% in the highest wealth index quartile. There was also a significant drop in retention after the institutional delivery for postnatal care. Overall, the retention of women belonging to the wealth index quartiles 1, 2, and 3 ranging from 2.9% to 5.5% was significantly lower than the 15.6% retention for the highest wealth index quartile.The inequality patterns in coverage of maternal care services are shown in Figure 2. The distance between the wealth index quartiles for four or more ANC visits shows the coverage to be similarly low in wealth index quartiles 1 to 3, but the wealthiest quartile was ahead of the rest (27.7%), suggesting a top inequality pattern for ANC coverage. The proportion of institutional delivery ranged from 49.5% for the lowest wealth index quartile to 77.7% for the wealthiest quartile. The coverage of postnatal care within 2 weeks of delivery was 21.2% for wealth index quartile 1 and 29.2% for quartile 2, and higher at 40.7% and 41.5% for quartiles 3 and 4, respectively. A pattern of mass deprivation was seen for coverage of four or more ANC visits, health facility delivery and postnatal care for the same woman. This proportion was close at 2.1-4.1% for the first three quartiles, and 12.2% even for the highest wealth index quartile.

## Discussion

Maternal and child health have been on the forefront of health policy in India for decades. The Government of India launched in early 2013 a national strategy to accelerate child survival and improve maternal health [27]. Efforts to improve the continuing poor state of maternal and child health in India require sub-national data as many states in India have more population than most countries of the world [18]. We report here a relatively high neonatal mortality rate in Bihar (which has over 104 million people and is one of the least developed states of India), associations with neonatal mortality, and an abysmally low retention of pregnant women in the health system for all components of maternal care.

Our estimate of NMR for the state of Bihar was 32.2 per 1,000 live births for reference year 2011, as data were collected in early 2012 for the past 12 months. The Annual Health Survey (AHS) reported an NMR of 34 per 1,000 live births in Bihar for reference years 2008–2010 [28], whereas the Sample Registration System (SRS) reported a relatively lower NMR of 29 per 1,000 live births in Bihar for 2011 [29]. AHS has larger sample size than SRS, and its NMR estimate is centred around 2009, is more consistent with our estimate of 32 per 1,000 live births in 2011 than is the lower NMR estimate for 2011 from SRS. The Global Burden of Disease Study 2013 has estimated the global neonatal mortality rate as 18.4 per 1000 live births for 188 countries [2]. While the neonatal mortality rates is highest in some of the countries in Sub–Saharan Africa, the rate of 28.1 per 1,000 live births for India also falls in the high end of the global distribution. It is particularly interesting to note that neighbouring Sri Lanka has a neonatal mortality rate of 4.5 per 1,000 live births, which is about one-sixth that of India. There is substantial variation in the neonatal mortality rates across India, for example, the rate of 39 per 1,000 live births in Madhya Pradesh state in north region and Odisha sate in east region is over five time higher than the rate of 7 in Kerala state of India in south region [29]. The overall neonatal mortality rate of 33 per 1,000 live births in rural India is twice the rate of 16 in urban India.

We found significant associations of neonatal mortality with postnatal care related variables. Not delaying bathing of new born and not providing kangaroo care immediately after birth were strongly associated with neonatal deaths. Postnatal care visits or examination and kangaroo care have been reported to have protective effect on new born [30–32]. Both delayed bathing and kangaroo care are included in the suggested indicators for tracking of new born care [33]. Although provision of postnatal care has been a component of various maternal and child health interventions in India; it has received less attention than skilled attendance at birth and ANC. Given the challenges in documenting postnatal care in household surveys, a standard set of questions have been suggested to assess postnatal care [33].

We found an association of neonatal mortality with not consuming adequate IFA tablets during pregnancy. IFA supplementation during pregnancy can reduce pre-term delivery, increase infant birth weight, prevent birth asphyxia and thereby reduce neonatal deaths [34, 35]. There is evidence from other studies also that supplementation of IFA tablets during pregnancy can reduce neonatal mortality in developing countries [36, 37]. Analysis of data from a national survey in India has reported a protective effect of two or more tetanus injections during pregnancy on neonatal deaths [38], but we did not find this association in our data.

Pregnancies with complications that included prolonged labor, excessive bleeding or convulsions had a higher risk of neonatal death in our study. Higher neonatal mortality with maternal complications has been previously documented in the literature [4]. Complications during labor are associated with greater risk of neonatal death than those identified during pregnancy [4, 39]. With obstructed labor and malpresentation carrying the highest risk which require skilled intervention, and in the background of prematurity, asphyxia and sepsis being the main causes of neonatal mortality in India [27, 40, 41], access to adequate obstetric and new born care is needed to reduce neonatal deaths in Bihar.

Neonatal deaths in our study were significantly higher for deliveries that occurred in private sector health facilities as compared with others. While the private sector facilities had a higher proportion of deliveries in women who reported prolonged labor, excessive bleeding or convulsions, the odds of neonatal deaths for deliveries in private facilities were higher even after adjusting for this variable. This suggests that there are other contributors to the higher neonatal mortality in private facilities, such as quality of care. Deliveries in private health facilities were associated with a much higher neonatal mortality among the two lower wealth index quartiles as compared with other deliveries. This difference was not observed for deliveries among the higher two wealth index quartiles. This finding indicates that the quality of private sector services being utilized by the lower socioeconomic strata in Bihar has significant deficiencies. There is perception in Bihar that some of the low-end private health providers conducting deliveries have totally inadequate facilities and skills. This aspect needs to be urgently and systematically assessed in order to prevent its continuing contribution to the poor neonatal outcomes in Bihar.

Neonatal mortality in our data from Bihar was highest for mothers in the 15–19 years age group. The recent approach outlined by the Government of India for continuum of care with a focus on various life stages including adolescence could assist with addressing health of adolescent girls which can impact pregnancy and health of the new born, in addition to delaying the age at marriage for girls [27]. The higher neonatal mortality in males in our data has also been reported in other studies [42, 43].

The overall utilization of maternal care was very poor in Bihar. It is important to note that only 5% of the pregnant women in Bihar received a combination of four or more ANC visits, institutional delivery and postnatal care within two weeks. Of the 73.8% women who started ANC visits, huge drops in proportion were observed between the first and fourth ANC visits as well as between institutional delivery and postnatal care. Of the women who started ANC, 84.8%, 39.6% and 19.3% went on to the second, third and fourth ANC visit, respectively. The extremely low utilization of the sequence of maternal care highlights that the maternal and child health interventions in Bihar will require a massive effort to provide adequate maternal care to the majority of mothers.

It is essential to monitor the coverage of health interventions in population subgroups as the national or sub-national averages can hide inequalities. The need to understand social and economic inequalities and disparities in health intervention coverage is also in line with the WHO Commission on the Social Determinants of Health and the recent report from Save the Children [6, 44]. The common measures of socioeconomic position used to assess inequality are education of the mother, income, consumption expenditure, and occupation with each having its own limitations [25]. We used a comprehensive composite wealth index made up of 33 assets to assess the pattern of inequalities for the various level of utilization of maternal health care services. Varying degrees of inequality was observed in our data from Bihar across the wealth index quartiles for four or more ANC visits, institutional delivery and postnatal care within 2 weeks. Therefore, while addressing the overall poor utilization of maternal care in Bihar, it would also be important to address the inequalities in the utilization of maternal care.

Overall, 14% pregnant women in Bihar had four or more ANC visits and 33.3% had postnatal care within two weeks, whereas a substantially higher proportion had institutional delivery (62.2%). This could be related to the monetary incentive given to pregnant women for having delivery in a health facility through the national *Janani Suraksha Yojana* (Safe Motherhood Scheme). Analysis of data from a previous nationwide survey has reported reduced neonatal mortality among women who availed this scheme [45]. It is possible that incentives for ANC and postnatal care could also increase their utilization. We suggest that it would be useful to consider a comprehensive incentive scheme that includes all three components of maternal care, i.e. ANC, institutional delivery and PNC. However, it is important to note that in the data presented in this paper, institutional deliveries were not associated with reduced neonatal mortality in Bihar as compared with home deliveries. On the other hand, deliveries in the private sector facilities were associated with much higher neonatal mortality among the lower two wealth index quartiles. This highlights the very crucial point that it is not enough to just focus on increasing utilization of services, but it is equally important to ensure adequate quality of those services.

Based on the analysis of trends by the Global Burden of Disease Study 2013, a substantial reduction in neonatal deaths is required in India to meet the MDG target of a two-thirds reduction in child mortality [2]. Similarly, a lot more progresses is needed to meet the MDG target of maternal mortality reduction in India [46]. The very poor overall utilization of maternal care and the relatively high neonatal mortality rate in Bihar state of India, as reported in this paper, emphasise the need for particular attention to achieving provision of maternal and newborn care to a much higher proportion of women than the present level. Also important in this effort would be to ensure adequate quality of care to achieve the desired outcome of reducing mortality.

One limitation of our data is that detailed data were available only for 63% of the neonatal deaths initially documented in listing, as nearly a quarter of the women with such deaths were away from their households for an extended period. Another limitation is that we were not able to explore relationships about the referrals between the public and private health facilities as these data were not available. In addition, variables such as birth weight and clean cord care have also been reported to influence neonatal survival [43, 47], but we did not have adequate data in our study to explore these associations. It is also possible that there could have been recall bias for some of the details of the maternal care that were documented in the interview.

Even with some limitations, the population-based data reported in this paper from across all districts of the Indian state of Bihar provide reasonably reliable estimates of neonatal mortality and its associations that should be generalizable. These findings can be utilized by health planners to improve maternal care utilization and reduce neonatal mortality. The analysis reported in this paper was carried out on data from the Ananya baseline data. Follow-up surveys have been planned. Subsequent comparisons with data from the follow-up surveys are likely to provide important insights into the trends of neonatal mortality and maternal care utilization in Bihar, and the influence of interventions and other variables on the changes that occur over time.

## Conclusions

Neonatal mortality is relatively high in Bihar and its associations include birth by younger mothers, lower socioeconomic strata, maternal complications during pregnancy, births in private health facilities, not receiving post-natal care, not providing skin-to-skin contact to the newborn soon after birth, and not delaying bathing of the newborn after birth. All socioeconomic strata in Bihar do poorly in complete utilization of maternal services, and therefore improvements are needed for most women in order to achieve optimal neonatal and maternal health outcomes. Inequality in the use of maternal care services between the wealth index strata in Bihar indicates that reduction in neonatal mortality could be facilitated further by better delivery of maternal care interventions, especially to the disadvantaged strata. The poor outcome associated with deliveries in private facilities points to the need for paying more attention to the quality of services.

## Electronic supplementary material

Additional file 1: **STROBE checklist of items for reports of cross-sectional studies.** (DOC 66 KB)

## Abbreviations

AHS: Annual Health Survey
ANC: Antenatal care
CI: Confidence interval
IFA: Iron folic acid
MDG: Millennium Development Goal
NMR: Neonatal mortality rate
OR: Odds ratio
SRS: Sample Registration System.
